# Association of Obsessive-Compulsive Disorder and Obsessive-Compulsive Symptoms With Substance Misuse in 2 Longitudinal Cohorts in Sweden

**DOI:** 10.1001/jamanetworkopen.2022.14779

**Published:** 2022-06-06

**Authors:** Suvi Virtanen, Ralf Kuja-Halkola, Anna Sidorchuk, Lorena Fernández de la Cruz, Christian Rück, Sebastian Lundström, Jaana Suvisaari, Henrik Larsson, Paul Lichtenstein, David Mataix-Cols, Antti Latvala

**Affiliations:** 1 Institute of Criminology and Legal Policy, University of Helsinki, Helsinki, Finland; 2 Department of Medical Epidemiology and Biostatistics, Karolinska Institutet, Stockholm, Sweden; 3 Centre for Psychiatry Research, Department of Clinical Neuroscience, Karolinska Institutet and Stockholm Health Care Services, Region Stockholm, Stockholm, Sweden; 4 Gillberg Neuropsychiatry Centre, University of Gothenburg, Gothenburg, Sweden; 5 Finnish Institute for Health and Welfare, Helsinki, Finland; 6 School of Medical Sciences, Örebro University, Örebro, Sweden

## Abstract

**Importance:**

Neurobiological models have postulated shared neural mechanisms between obsessive-compulsive disorder (OCD) and substance use disorders, but results from clinical and epidemiological studies are conflicting or even suggest that OCD may be protective against substance misuse.

**Objective:**

To investigate whether OCD and obsessive-compulsive symptoms are associated with substance misuse and the extent to which shared genetic and/or environmental factors account for this association.

**Design, Setting, and Participants:**

In this cohort study, individuals in the general population of Sweden born between January 1, 1932, and December 31, 1997 (population cohort), were followed up through Swedish nationwide registers from January 1, 1997, to December 31, 2013. The second cohort included twin participants in the Child and Adolescent Twin Study in Sweden (CATSS) followed up from ages 18 to 24 years. Data were analyzed from March 1, 2021, to March 31, 2022.

**Exposures:**

Lifetime *International Statistical Classification of Diseases and Related Health Problems, Tenth Revision*, diagnosis of OCD in the National Patient Register (population cohort 1), and self-reported obsessive-compulsive symptoms at 18 years of age (CATSS cohort).

**Main Outcomes and Measures:**

Substance misuse was defined as registered substance use–related disorder, criminal conviction, or death (population cohort), and self-reported alcohol and drug dependence symptoms at 18 and 24 years of age (CATSS cohort).

**Results:**

The general population cohort included 6 304 188 individuals (48.9% women and 51.1% men; median baseline age, 30.5 [IQR, 15.0-46.4] years), of whom 27 342 had an OCD diagnosis. Obsessive-compulsive disorder was associated with an elevated risk of substance misuse (hazard ratio, 3.68 [95% CI, 3.52-3.85]). In the 9230 individuals in the CATSS cohort (5551 women [60.1%] and 3679 men [39.9%]), obsessive-compulsive symptoms at 18 years of age were associated with increased symptoms of alcohol dependence (concurrent [n = 9219], β = 0.18 [95% CI, 0.16-0.20]; longitudinal [n = 3381], β = 0.10 [95% CI, 0.06-0.14]) and drug dependence (concurrent [n = 749], β = 0.19 [95% CI, 0.11-0.27]; longitudinal [n = 452], β = 0.15 [95% CI, 0.04-0.25]). Comorbid anxiety and depression did not entirely explain the associations in either cohort. Using data from full siblings and maternal half-siblings (population cohort) and monozygotic and dizygotic twins (CATSS cohort) provided estimates of the relative contribution of genetic and environmental influences to the covariance between OCD and obsessive-compulsive symptoms and substance misuse or dependence. The associations were explained by genetic (56%-68%) and nonshared environmental (32%-44%) factors.

**Conclusions and Relevance:**

The findings of this Swedish population-based cohort study challenge the notion that OCD is protective against developing substance misuse. The association of OCD and obsessive-compulsive symptoms with substance misuse was largely explained by shared genetics but was also compatible with partial environmental mediation.

## Key Points

**Question:**

Do individuals with obsessive-compulsive disorder (OCD) or obsessive-compulsive symptoms have an elevated risk of substance misuse, and if so, to what extent do shared genetic and/or environmental factors account for their association?

**Findings:**

This Swedish cohort study of 6 304 188 individuals from the general population and 9230 individuals in a separate twin cohort found that individuals with an OCD diagnosis had a 3.7-fold elevated risk of any substance misuse outcome. The association of OCD and obsessive-compulsive symptoms with substance misuse was partially attributed to shared genetics.

**Meaning:**

These findings suggest that regular screening for substance use and problems should be included in routine clinical management of patients with OCD.

## Teaser

This cohort study assesses the association of obsessive-compulsive disorder and obsessive-compulsive symptoms with substance misuse in a general population sample and a twin sample in Sweden.

## Introduction

Neurobiological models of obsessive-compulsive disorder (OCD) postulate the involvement of several parallel, partly segregated, cortico-striato-thalamo-cortical circuits.^1,2,3^ Among these, a ventral affective circuit, which includes the orbitofrontal cortex, nucleus accumbens, and thalamus, is thought to be involved in reward processing.^1,2,3^ Consequently, neurobiological models predict an association of OCD with substance use disorders (SUDs) and behavioral addictions,^4,5,6^ possibly reflecting a shared compulsivity endophenotype.^7,8,9^ However, empirical support for this predictive model has been mixed.

Remarkably, whether individuals with OCD have higher prevalence of SUDs compared with the general population remains unclear. Studies with clinical samples^10,11,12,13,14^ have reported SUD prevalence similar to that of the general population. Other studies^15,16,17^ have even suggested that people with OCD might be less likely to use substances owing to risk aversion. In contrast, epidemiological surveys^18,19,20,21,22^ have found an elevated risk of SUDs in individuals with OCD. However, many clinical studies did not include a control group, and epidemiological surveys often had few cases with OCD, resulting in imprecise estimates. Moreover, the lack of longitudinal and genetically informative data has hindered progress in this field.

The heritability of both OCD and SUDs is well established,^23,24,25^ but studies are only beginning to clarify the contribution of familial influences to their association. Although some studies found no familial coaggregation,^10,11^ an elevated risk of OCD among first-degree relatives of individuals with alcohol dependence has been reported.^26^ A study using genome-wide association study (GWAS) summary statistics found a statistically nonsignificant negative genetic correlation between OCD and SUDs,^27^ whereas the latest GWAS of OCD shows a positive genetic correlation with alcohol dependence.^28^ Theoretically, a shared endophenotype for OCD and SUDs should manifest as a positive genetic correlation between the two. On the other hand, a direct effect of OCD on SUDs (eg, via self-medication) would be detected as a nonshared environmental correlation, independent of shared genetics. Quantitative genetic studies can test these competing hypotheses, but to our knowledge, no prior sibling or twin studies exist.

We studied the association of OCD with substance misuse in 2 population-based cohorts. First, we investigated associations between clinician-diagnosed OCD and substance misuse–related outcomes in the Swedish general population. Second, using a large twin sample, we studied whether self-reported obsessive-compulsive symptoms were associated with concurrent and subsequent alcohol and drug dependence symptoms among young adults. In both cohorts, we examined whether the associations were explained by anxiety and depression and estimated the relative contribution of genetic and environmental influences to the covariance between OCD and obsessive-compulsive symptoms and substance misuse.

## Methods

### Data

The Regional Ethical Review Board in Stockholm approved the register linkage for the population-based cohort. Informed consent was not required because the data have been anonymized. The Child and Adolescent Twin Study in Sweden (CATSS) was approved by the Regional Ethics Review Board in Stockholm. The participants provided informed consent. This study was reported according to the Strengthening the Reporting of Observational Studies in Epidemiology (STROBE) reporting guideline.

#### Population Cohort

The population cohort included people born in Sweden between January 1, 1932, and December 31, 1997, excluding those who died or emigrated before the start of follow-up (n = 445 893). A total of 6 304 188 individuals were linked to nationwide registers via the personal identity number assigned to Swedish residents.^29^ We used the National Patient Register (NPR),^30^ the Crime Register,^31^ the Cause of Death Register,^32^ the Migration Register,^33^ and the Multi-Generation Register, which allows for linking individuals with their parents.^34^ The cohort was followed up from January 1, 1997, or the 15th birthday, whichever occurred last, until the date of outcome, first emigration, death, or December 31, 2013, whichever occurred first.

We identified full siblings and maternal half-siblings within the cohort. From each sibling cluster, we selected first- and second-born siblings whose age difference was not more than 5 years. Adoptees and twins were excluded. This subcohort included 3 317 168 full siblings and 130 256 half-siblings. The same person could appear in a full-sibling and a half-sibling pair.

#### Twin Cohort

The CATSS is an ongoing longitudinal study targeting all twins born in Sweden since July 1, 1992.^35^ Our sample consisted of participants (born January 1, 1993, to December 31, 2001) who provided information on OCD, anxiety, depression, and substance misuse symptoms at 18 years of age and who endorsed using alcohol or drugs (n = 9230). Participants with missing values were excluded (n = 3473). A subset of the sample had follow-up data at 24 years of age. Those who endorsed using alcohol or drugs were included (n = 3414). Participants who had not used alcohol or drugs at 18 years of age but reported use at 24 years of age (n = 426) were also included in the age 24 sample.

### Measures

#### Population Cohort

##### Exposure: OCD

We collected *International Statistical Classification of Diseases and Related Health Problems, Tenth Revision* (*ICD-10*), diagnoses of OCD (code F42) from the NPR. The NPR covers *ICD-10* diagnoses from psychiatric inpatient and outpatient specialist services since 1997 and 2001, respectively. Diagnoses registered before 6 years of age were excluded to avoid diagnostic misclassification. The *ICD-10* codes for OCD in the NPR have excellent validity and reliability.^36^ The date of the first OCD diagnosis was used as a time-varying exposure.

##### Outcomes: Substance Use–Related Disorders, Criminal Convictions, and Deaths

We defined substance-related disorders as any *ICD-10* code for SUDs, alcohol- and/or drug-related somatic conditions, or poisoning by alcohol and/or drugs registered between January 1, 1997, and December 31, 2013, in the NPR (eTable 1 in the Supplement). Alcohol-related disorders were defined in accordance with previously established guidelines.^37^ To improve validity, we excluded registrations before 15 years of age. We retrieved alcohol- and drug-related deaths from the Cause of Death Register (eTable 1 in the Supplement). Substance use–related convictions in the Crime Register included driving under the influence of alcohol and drugs. The age for criminal responsibility in Sweden is 15 years.

##### Covariates: Psychiatric Disorders

A priori covariates were comorbidity with lifetime diagnoses of anxiety (*ICD-10* code F40-F41) and depressive (*ICD-10* codes F32-F34 and F38-F39, excluding F34.0) disorders in the NPR. In post hoc analyses, we also included other relevant psychiatric disorders (eMethods in the Supplement).

#### Twin Cohort

##### Exposure: Obsessive-Compulsive Symptoms

At 18 years of age, the twin participants completed the Brief Obsessive-Compulsive Scale,^38^ which is based on the clinician-administered Yale-Brown Obsessive-Compulsive Scale. Participants rated each of its 15 items as never, past, or current. In accordance with previous studies,^39^ we combined the 2 endorsing categories, coding past and current as 1 and never as 0, and excluded 3 items related to hoarding, dysmorphia, and self-harm because they do not represent the core OCD phenotype. The 12-item scale had good internal consistency (Cronbach α = 0.76).

##### Outcomes: Substance Use and Dependence Symptoms

Alcohol use problems were measured with the self-reported Alcohol Use Disorders Identification Test^40^ at 18 and 24 years of age. The Alcohol Use Disorders Identification Test is a 10-item scale; items 1 to 3 relate to consumption of alcohol (frequency and quantity) and items 4 to 10 measure alcohol dependence and harmful use (ie, loss of control, withdrawal, neglect of other pursuits, continued use despite harm). Previous research supports a 2-factor structure, where items 1 to 3 load on factor 1 and items 4 to 10 load on factor 2.^41^ We identified individuals who used alcohol based on item 1 of the Alcohol Use Disorders Identification Test. The outcome variable, alcohol dependence symptoms, was measured as the sum of items 4 to 10. Internal consistency of the scale was Cronbach α = 0.67 at 18 years of age and Cronbach α = 0.70 at 24 years of age.

Drug use problems were measured with the self-reported Drug Use Disorders Identification Test^42^ at 18 and 24 years of age. The Drug Use Disorders Identification Test consists of 11 items, with items 1 to 4 measuring frequency and quantity of drug use and items 5 to 11 relating to dependence and harm. Validation studies have identified a 2-factor structure, with factors representing (1) drug consumption and (2) dependence and harmful consequences.^43^ We identified participants who used drugs based on item 1, and measured drug dependence symptoms as the sum of items 5 to 11. Internal consistency of the scale was Cronbach α = 0.74 at 18 years of age and Cronbach α = 0.83 at 24 years of age.

##### Covariates: Anxiety and Depressive Symptoms

Anxiety was measured at 18 years of age with the self-reported Screen for Child Anxiety Related Emotional Disorders.^44^ The Screen for Child Anxiety Related Emotional Disorders is a validated,^44,45,46^ 38-item symptom checklist with items reflecting common anxiety diagnoses. Items are scored on a 3-point scale. Internal consistency of the scale was Cronbach α = 0.93.

Depressive symptoms were measured at 18 years of age with a self-reported version of the Center for Epidemiologic Studies Depression Scale,^47^ which includes 11 items scored on a 4-point scale. The scale has good psychometric properties.^48^ Internal consistency of the scale was Cronbach α = 0.87.

### Statistical Analysis

#### Phenotypic Associations

Data were analyzed from March 1, 2021, to March 31, 2022. In the population cohort, we used Cox proportional hazards regression, with age as the underlying timescale, to estimate the association between OCD and substance misuse outcomes. We present estimates for the full sample and separately for men and women. Further, we estimated the cumulative incidence of any alcohol- or drug-related disorder for individuals with and without a lifetime diagnosis of OCD using Kaplan-Meier survival estimates under the assumption of no competing risks. The Kaplan-Meier estimation was restricted to individuals 15 years or younger in 1997 (n = 1 623 889). To test whether the association between OCD and substance misuse was explained by comorbid anxiety and depressive disorders, the analyses were repeated adjusting for these diagnoses. For a post hoc analysis, we also adjusted for other psychiatric comorbidities (eMethods in the Supplement). All models were adjusted for sex and birth year.

In the CATSS cohort, we used linear regression to estimate concurrent and longitudinal associations between obsessive-compulsive symptoms and alcohol and drug dependence symptoms. Concurrent associations were estimated by regressing dependence symptoms at 18 years of age on obsessive-compulsive symptoms at 18 years of age. For longitudinal associations, dependence symptoms at 24 years of age were regressed on obsessive-compulsive symptoms in participants at 18 years of age with complete data at 24 years of age. Both models were also estimated adjusting for anxiety and depressive symptoms at 18 years of age. Finally, dependence symptoms at 24 years of age were regressed on obsessive-compulsive symptoms with adjustment for dependence symptoms at 18 years of age. Exposure and outcome variables were standardized. All models adjusted for sex and birth year. We used cluster-robust SEs to account for clustering of observations. Analyses were conducted with Stata, version 17 (StataCorp LLC.). 

#### Quantitative Genetic Models

We conducted quantitative genetic modeling to estimate the contribution of genetic and environmental factors to the associations of lifetime OCD with any substance misuse (population cohort) and obsessive-compulsive symptoms with alcohol dependence symptoms at 18 years of age (CATSS cohort). We used the full-sibling and maternal half-sibling design in the population cohort and the classical twin design in the CATSS cohort. Both designs rely on assumptions concerning genetic and environmental sharing within twin and sibling pairs: monozygotic twins are genetically identical, whereas dizygotic twins share approximately 50% of their segregating genes. Similarly, full siblings share 50% of their genes, whereas maternal half-siblings share approximately 25%. All sibling types are assumed to share environments with their cosibling equally. Using structural equation modeling, the variance of a phenotype and the covariance between phenotypes was decomposed into latent additive genetic (A), shared environmental (C), and nonshared environmental factors (E). We also calculated genetic and environmental correlations (ie, the correlation of genetic and environmental variance components between the 2 traits). We used the direct-symmetric parameterization, which lowers the risk for type I errors, but can produce negative variance contributions.^49^ For dichotomous variables, we used a liability-threshold model where the categories (lifetime diagnosis of OCD and substance misuse present vs not present) were assumed to reflect an underlying normal distribution of liability.^50^ Models were adjusted for sex and birth year. The model with fewer parameters was considered to have the best fit if not significantly worse than the full ACE-ACE model (including A, C, and E components for both phenotypes), as indicated by the Akaike information criterion and *P* value for the reduction of model fit in the likelihood ratio test. Two-sided *P* < .05 indicated statistical significance. Analyses were conducted with R, version 4.0.5 (R Core Team).

## Results

### Phenotypic Associations

#### Population Cohort

The cohort included 6 304 188 individuals (48.9% women and 51.1% men; median age, 30.5 [IQR, 15.0-46.4] years) with a median length of follow-up of 16.9 (IQR, 11.2-16.9) years. Altogether 27 342 individuals had an OCD diagnosis (57.2% women and 42.9% men). The median age at first OCD diagnosis was 28.0 (IQR, 20.3-39.8) years. Cohort characteristics are presented in eTable 2 in the Supplement.

Obsessive-compulsive disorder was associated with an elevated risk of all substance misuse outcomes compared with individuals without OCD (Table 1), specifically, a 4.5-fold increased risk of alcohol-related disorder (hazard ratio [HR], 4.51 [95% CI, 4.25-4.79]), 6.7-fold increased risk of any drug-related disorder (HR, 6.69 [95% CI, 6.33-7.07]), 1.2-fold increased risk of substance use–related criminal conviction (HR, 1.24 [95% CI, 1.09-1.41]), and 5.2-fold increased risk of substance use–related death (HR, 5.20 [95% CI, 4.45-6.08]). The risk of sedative- and other drug–related disorders was particularly elevated, with 10.5-fold (HR, 10.53 [95% CI, 9.84-11.28]) and 6.3-fold (HR, 6.32 [95% CI, 5.86-6.81]) increased risks, respectively, compared with individuals without OCD.

**Table 1.** Association of Obsessive-Compulsive Disorder With Substance Misuse Outcomes by Sex in the Population Cohort

Outcome	Individuals, No. (%)		HR (95% CI)	
	With OCD	Unaffected general population	Adjusted for sex and birth year	Adjusted for sex, birth year, and anxiety and depressive disorders
All	27 342 (100)	6 276 846 (100)	NA	NA
Men	11 717 (100)	3 212 821 (100)	NA	NA
Women	15 625 (100)	3 064 025 (100)	NA	NA
Any substance misuse outcome	5444 (19.9)	359 393 (5.7)	3.68 (3.52-3.85)	1.10 (1.05-1.15)
Men	2571 (21.9)	247 188 (7.7)	2.76 (2.58-2.95)	0.91 (0.86-0.98)
Women	2873 (18.4)	112 205 (3.7)	4.92 (4.64-5.23)	1.30 (1.22-1.38)
Alcohol-related disorders	2720 (9.9)	163 591 (2.6)	4.51 (4.25-4.79)	1.10 (1.04-1.17)
Men	1449 (12.4)	112 223 (3.5)	4.01 (3.69-4.36)	1.00 (0.92-1.09)
Women	1271 (8.1)	51 368 (1.7)	4.99 (4.58-5.43)	1.19 (1.09-1.30)
Acute alcohol intoxications	1577 (5.8)	90 351 (1.4)	3.64 (3.38-3.93)	1.13 (1.05-1.22)
Men	716 (6.1)	54 203 (1.7)	3.43 (3.08-3.83)	1.08 (0.97-1.21)
Women	861 (5.5)	36 148 (1.2)	3.80 (3.43-4.22)	1.17 (1.06-1.30)
Any drug-related disorders	3279 (12.0)	100 165 (1.6)	6.69 (6.33-7.07)	1.28 (1.22-1.36)
Men	1434 (12.2)	57 595 (1.8)	5.76 (5.30-6.25)	1.11 (1.02-1.20)
Women	1845 (11.8)	42 570 (1.4)	7.94 (7.37-8.55)	1.52 (1.41-1.64)
Opioid-related disorders	618 (2.3)	21 810 (0.3)	6.19 (5.52-6.95)	1.22 (1.09-1.37)
Men	280 (2.4)	12 986 (0.4)	5.79 (4.90-6.83)	1.10 (0.93-1.29)
Women	338 (2.2)	8824 (0.3)	6.79 (5.78-7.97)	1.39 (1.18-1.63)
Cannabis-related disorders	448 (1.6)	17 410 (0.3)	3.72 (3.31-4.32)	0.87 (0.75-1.01)
Men	300 (2.6)	13 396 (0.4)	3.57 (2.98-4.28)	0.83 (0.69-0.99)
Women	148 (0.9)	4014 (0.1)	4.20 (3.24-5.45)	1.00 (0.77-1.30)
Sedative-related disorders	1942 (7.1)	44 013 (0.7)	10.53 (9.84-11.28)	1.72 (1.60-1.84)
Men	734 (6.3)	20 411 (0.6)	10.52 (9.45-11.70)	1.58 (1.42-1.76)
Women	1208 (7.7)	23 602 (0.8)	10.82 (9.90-11.82)	1.86 (1.70-2.03)
Stimulant-related disorders	495 (1.8)	18 999 (0.3)	4.89 (4.26-5.61)	1.06 (0.93-1.22)
Men	244 (2.1)	12 929 (0.4)	3.77 (3.08-4.62)	0.80 (0.65-0.98)
Women	251 (1.6)	6070 (0.2)	6.55 (5.43-7.90)	1.50 (1.24-1.81)
Other drug-related disorders	1620 (5.9)	49 547 (0.8)	6.32 (5.86-6.81)	1.20 (1.11-1.29)
Men	801 (6.8)	32 002 (1.0)	5.27 (4.73-5.87)	0.98 (0.88-1.09)
Women	819 (5.2)	17 545 (0.6)	7.84 (7.08-8.70)	1.52 (1.37-1.69)
Substance-related convictions	697 (2.5)	122 703 (2.0)	1.24 (1.09-1.41)	0.54 (0.47-0.62)
Men	511 (4.4)	106 848 (3.3)	1.05 (0.90-1.22)	0.47 (0.40-0.55)
Women	186 (1.2)	15 855 (0.5)	2.13 (1.69-2.69)	0.77 (0.61-0.97)
Substance-related deaths	161 (0.6)	27 479 (0.4)	5.20 (4.45-6.08)	2.33 (1.91-2.61)
Men	105 (0.9)	21 317 (0.7)	4.62 (3.81-5.61)	2.10 (1.72-2.55)
Women	56 (0.34)	6165 (0.2)	6.87 (5.27-8.95)	2.58 (1.98-3.37)

Abbreviations: HR, hazard ratio; NA, not applicable; OCD, obsessive-compulsive disorder.

As shown in the Figure, the cumulative incidence of alcohol- and drug-related disorders in individuals with OCD was 23% by 32 years of age (5% in the general population). Differences between groups were evident by 16 years of age (based on nonoverlapping 95% CIs).

**Figure.**
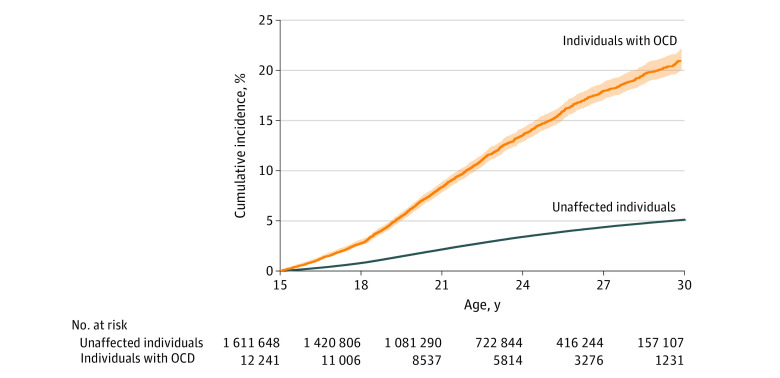
Cumulative Incidence of Any Alcohol- or Drug-Related Disorders in the Population Cohort Calculations were performed under the assumption of no competing risks (estimated as 1 minus the Kaplan-Meier estimate of survival function with 95% CIs) among individuals with a lifetime diagnosis of obsessive-compulsive disorder (OCD) and unaffected individuals from the general population. The analysis was restricted to individuals with follow-up from 15 years or younger (n = 1 623 889).

When adjustments were made for anxiety and depressive disorders, all associations greatly attenuated (Table 1). Most of the associations remained elevated even after adjustment, except for cannabis (HR, 0.87 [95% CI, 0.75-1.01]), stimulants (HR, 1.06 [95% CI, 0.93-1.22]), and substance-related criminal convictions (HR, 0.54 [95% CI, 0.47-0.62]). A post hoc analysis adjusting for other psychiatric comorbidities attenuated the associations but did not fully explain them, except in substance-related convictions (HR excluding attention-deficit/hyperactivity disorder, 0.77 [95% CI, 0.67-0.87]; HR excluding bipolar disorder, 1.04 [95% CI, 0.92-1.19]; HR excluding psychotic disorders, 1.04 [95% CI, 0.91-1.18]; HR excluding personality disorders, 0.96 [95% CI, 0.84-1.09]) (eTable 3 in the Supplement).

#### Twin Cohort

Descriptive statistics of the 9230 individuals in the CATSS cohort (5551 women [60.1%] and 3679 men [39.9%]) are presented in eTable 4 in the Supplement. Obsessive-compulsive symptoms were associated with increased symptoms of alcohol and drug dependence (Table 2). Among people who reported alcohol and drug use, a 1-SD increase in obsessive-compulsive symptoms was associated with a 0.2-SD increase in concurrent dependence symptoms (for alcohol use [n = 9219], β = 0.18 [95% CI, 0.16-0.20]; for drug use [n = 749], β = 0.19 [95% CI, 0.11-0.27]). Similar associations were observed in the longitudinal analyses (for alcohol use [n = 3381], β = 0.10 [95% CI, 0.06-0.14]; for drug use [n = 452], β = 0.15 [95% CI, 0.04-0.25]).

**Table 2.** Association of Obsessive-Compulsive Symptoms at 18 Years of Age With Substance Dependence Symptoms in the Child and Adolescent Twin Study in Sweden Participants Using Alcohol or Drugs

Symptom type by age	Exposure: obsessive-compulsive symptoms at 18 y, β (95% CI)		
	Adjusted for sex and birth year	Adjusted for sex, birth year, and anxiety and depression at 18 y	Adjusted for sex, birth year, and dependence symptoms at 18 y
**18 y**			
Alcohol dependence among individuals using alcohol (n = 9219)	0.18 (0.16-0.20)	0.12 (0.09-0.14)	NA
Drug dependence among individuals using drugs (n = 749)	0.19 (0.11-0.27)	0.12 (0.04-0.20)	NA
**24 y**			
Alcohol dependence among individuals using alcohol (n = 3381)	0.10 (0.06-0.14)^a^	0.05 (0.00-0.09)^a^	0.05 (0.01-0.09)^b^
Drug dependence among individuals using drugs (n = 452)	0.15 (0.04-0.25)	0.11 (0.00-0.22)	NA^c^

Abbreviation: NA, not applicable.

^a^ Includes 3389 participants.

^b^ Includes 2965 participants.

^c^ Model was not estimated owing to insufficient sample size.

After adjusting for anxiety and depressive symptoms, obsessive-compulsive symptoms were still associated with symptoms of alcohol dependence, both concurrently (for alcohol use, β = 0.12 [95% CI, 0.09-0.14]; for drug use, β = 0.12 [95% CI, 0.04-0.20]) and longitudinally (for alcohol use, β = 0.05 [95% CI, 0.00-0.09]; for drug use, β = 0.11 [95% CI, 0.00-0.22]). Further, obsessive-compulsive symptoms remained associated with alcohol dependence symptoms at 24 years of age when we accounted for alcohol dependence symptoms at 18 years of age (β = 0.05 [95% CI, 0.01-0.09]).

### Quantitative Genetic Analyses

In the population cohort, the phenotypic correlation between OCD and substance misuse was 0.27. The best-fitting model included A (explaining 55% of the variance) and E (explaining 45% of the variance) components for OCD and A (57%) and E (43%) components for substance misuse (eTables 5 and 6, model B, in the Supplement). Additive genetic factors explained 56% of the covariance between OCD and substance misuse, whereas nonshared environmental factors explained 44%. The estimated genetic correlation between the phenotypes was 0.28 (95% CI, 0.24-0.32), and the nonshared environmental correlation was 0.27 (95% CI, 0.22-0.32).

In the twin cohort, the phenotypic correlation between obsessive-compulsive symptoms and alcohol dependence symptoms was 0.19. The best-fitting model included A (explaining 36% of the variance) and E (explaining 64% of the variance) components for OCD symptoms, and A (explaining 46% of the variance) and E (explaining 54% of the variance) components for alcohol dependence symptoms (eTables 7 and 8, model B, in the Supplement). Additive genetic factors explained 68% of the covariance, and nonshared environmental influences explained the remaining 32%. The estimated genetic correlation was 0.31 (95% CI, 0.23-0.40), and the nonshared environmental correlation was 0.10 (95% CI, 0.05-0.16).

## Discussion

In 2 large, prospective cohorts, we found OCD and obsessive-compulsive symptoms to be associated with an elevated risk of substance misuse, corroborating findings from earlier cross-sectional surveys.^18,19,20,21,22^ Specifically, OCD was associated with a 4.5-fold increased risk of alcohol-related disorders, a 6.7-fold increased risk of any drug-related disorder, a 1.2-fold increased risk of substance use–related criminal conviction, and a 5.2-fold increased risk of substance use–related death compared with individuals without OCD. The higher incidence of alcohol- and drug-related disorders was already evident by 16 years of age. We found similar associations in the nonclinical CATSS cohort: in 18-year-old participants, obsessive-compulsive symptoms were associated with increased alcohol and drug dependence symptoms, both concurrently and longitudinally. Anxiety and depression contributed to the associations but did not fully account for them. Our results suggest that in contrast to earlier clinical studies, OCD is associated with at least similarly elevated risk of substance misuse, as are other common mental disorders.^50,51,52,53,54^ Regular screening for substance use should therefore be included in routine clinical management of patients with OCD, even in children and adolescents.

Consistent with the hypothesis of a shared endophenotype for OCD and substance misuse,^7,8,9^ we found shared genetic factors to explain approximately 56% to 68% of the covariance. The validity of our results was increased by the use of 2 separate cohorts and study designs with different assumptions. However, a genetic correlation by itself is insufficient to confirm the hypothesis because it might reflect some other genetically influenced trait instead of the hypothesized compulsivity endophenotype. A genetic correlation might reflect vertical pleiotropy (ie, phenotypic mediation) instead of shared genetic etiology.^55^ These hypotheses can be tested in future OCD GWAS benefiting from a substantially increased sample size^28^ compared with previous efforts.^56^

A third major finding was that the association of OCD with substance misuse was not entirely explained by genetic factors. A nonshared environmental correlation is compatible with an environmentally mediated relationship between the disorders, such as self-medication. However, our study cannot unequivocally confirm the self-medication hypothesis. We found a particularly high risk of sedative-related disorders in individuals with OCD, which warrants further investigation. It is possible that people with OCD are initially prescribed sedatives, eventually leading to misuse or, alternatively, that sedatives are acquired from other sources. Together, these results indicate that adequate management of obsessive-compulsive symptoms might be associated with a reduction of substance misuse.^57^ Interestingly, some evidence suggests that selective serotonin reuptake inhibitors, which constitute the first-line pharmacological treatment for OCD, could help reduce substance misuse among patients with comorbid anxiety and/or depression and SUDs,^58,59,60^ but further research is needed.

### Limitations

Our study had several limitations. First, register data do not capture all individuals with OCD or substance misuse because the NPR does not include diagnoses from primary care or private clinics, and some people never seek treatment. Thus, diagnoses in the population cohort represent a select group of treatment-seeking individuals requiring specialist treatment. This limitation was partially mitigated with the inclusion of CATSS data, which better captures individuals with less severe psychopathology. The consistency of findings based on register and twin data increases confidence in the validity of our results. However, although there is wide consensus that self-reported OCD symptoms in nonclinical samples reflect milder variants of those observed among individuals with OCD,^61^ they also likely capture a broader phenotype. Second, we cannot exclude the possibility of a reverse association in our data. Nevertheless, there is little evidence of substance-induced OCD in the scientific literature. Third, the associations may have been inflated by common method bias. This limitation was somewhat mitigated by the inclusion of information from the Crime Register and the Cause of Death Register, which are independent from the NPR.

## Conclusions

In this Swedish population-based cohort study, OCD and obsessive-compulsive symptoms were associated with an elevated risk of substance misuse, challenging the notion that OCD is protective against developing substance misuse. The associations of OCD and obsessive-compulsive symptoms with substance misuse were largely explained by shared genetics, but our findings are also compatible with an environmentally mediated relationship (eg, self-medication hypothesis).

## Notes

Supplement. **eMethods.** Post hoc Comorbidity Analyses **eTable 1.** *ICD-10* Codes for Substance Use-Related Disorders and Deaths **eTable 2.** Population Cohort Characteristics **eTable 3.** Post hoc Analyses: Association of Obsessive-Compulsive Disorder With Substance Misuse Outcomes, Adjusted for Psychiatric Comorbidities **eTable 4.** Descriptive Statistics of the Child and Adolescent Twin Study in Sweden (CATSS) Twin Cohort **eTable 5.** Population Cohort: Fit Statistics From the Quantitative Genetic Models **eTable 6.** Population Cohort: Parameter Estimates (95% CIs) From the Full ACE-ACE Model and the Best-Fitting Bivariate Model **eTable 7.** CATSS Cohort: Fit Statistics From the Quantitative Genetic Models **eTable 8.** CATSS Cohort: Parameter Estimates (95% CIs) From the Full ACE-ACE Model and the Best-Fitting Bivariate Model

## References

[1] van den Heuvel OA, van Wingen G, Soriano-Mas C, . Brain circuitry of compulsivity. Eur Neuropsychopharmacol. 2016;26(5):810-827. doi:10.1016/j.euroneuro.2015.12.005 26711687

[2] Shephard E, Stern ER, van den Heuvel OA, . Toward a neurocircuit-based taxonomy to guide treatment of obsessive-compulsive disorder. Mol Psychiatry. 2021;26(9):4583-4604. doi:10.1038/s41380-020-01007-8 33414496 PMC8260628

[3] Voon V, Baek K, Enander J, . Motivation and value influences in the relative balance of goal-directed and habitual behaviours in obsessive-compulsive disorder. Transl Psychiatry. 2015;5(11):e670. doi:10.1038/tp.2015.165 26529423 PMC5068758

[4] Figee M, Pattij T, Willuhn I, . Compulsivity in obsessive-compulsive disorder and addictions. Eur Neuropsychopharmacol. 2016;26(5):856-868. doi:10.1016/j.euroneuro.2015.12.003 26774279

[5] Robbins TW, Vaghi MM, Banca P. Obsessive-compulsive disorder: puzzles and prospects. Neuron. 2019;102(1):27-47. doi:10.1016/j.neuron.2019.01.046 30946823

[6] Yücel M, Lee RSC, Fontenelle LF. A new consensus framework for phenotyping and treatment selecting in addiction and obsessive-compulsive-related disorders. JAMA Psychiatry. 2021;78(7):699-700. doi:10.1001/jamapsychiatry.2021.0243 33825819

[7] Robbins TW, Gillan CM, Smith DG, de Wit S, Ersche KD. Neurocognitive endophenotypes of impulsivity and compulsivity: towards dimensional psychiatry. Trends Cogn Sci. 2012;16(1):81-91. doi:10.1016/j.tics.2011.11.009 22155014

[8] Voon V, Derbyshire K, Rück C, . Disorders of compulsivity: a common bias towards learning habits. Mol Psychiatry. 2015;20(3):345-352. doi:10.1038/mp.2014.44 24840709 PMC4351889

[9] Ersche KD, Jones PS, Williams GB, Smith DG, Bullmore ET, Robbins TW. Distinctive personality traits and neural correlates associated with stimulant drug use versus familial risk of stimulant dependence. Biol Psychiatry. 2013;74(2):137-144. doi:10.1016/j.biopsych.2012.11.016 23273722 PMC3705207

[10] Bienvenu OJ, Samuels JF, Wuyek LA, . Is obsessive-compulsive disorder an anxiety disorder, and what, if any, are spectrum conditions? a family study perspective. Psychol Med. 2012;42(1):1-13. doi:10.1017/S0033291711000742 21733222 PMC10885736

[11] Nestadt G, Samuels J, Riddle MA, . The relationship between obsessive-compulsive disorder and anxiety and affective disorders: results from the Johns Hopkins OCD Family Study. Psychol Med. 2001;31(3):481-487. doi:10.1017/S0033291701003579 11305856

[12] Denys D, Tenney N, van Megen HJ, de Geus F, Westenberg HG. Axis I and II comorbidity in a large sample of patients with obsessive-compulsive disorder. J Affect Disord. 2004;80(2-3):155-162. doi:10.1016/S0165-0327(03)00056-9 15207928

[13] Lochner C, Fineberg NA, Zohar J, . Comorbidity in obsessive-compulsive disorder (OCD): a report from the International College of Obsessive-Compulsive Spectrum Disorders (ICOCS). Compr Psychiatry. 2014;55(7):1513-1519. doi:10.1016/j.comppsych.2014.05.020 25011690

[14] Yaryura-Tobias JA, Grunes MS, Todaro J, McKay D, Neziroglu FA, Stockman R. Nosological insertion of axis I disorders in the etiology of obsessive-compulsive disorder. J Anxiety Disord. 2000;14(1):19-30. doi:10.1016/S0887-6185(99)00027-4 10770233

[15] Bejerot S, Humble M. Low prevalence of smoking among patients with obsessive-compulsive disorder. Compr Psychiatry. 1999;40(4):268-272. doi:10.1016/S0010-440X(99)90126-8 10428185

[16] Fineberg NA, Hengartner MP, Bergbaum C, Gale T, Rössler W, Angst J. Lifetime comorbidity of obsessive-compulsive disorder and sub-threshold obsessive-compulsive symptomatology in the community: impact, prevalence, socio-demographic and clinical characteristics. Int J Psychiatry Clin Pract. 2013;17(3):188-196. doi:10.3109/13651501.2013.777745 23428236

[17] Cuzen NL, Stein DJ, Lochner C, Fineberg NA. Comorbidity of obsessive-compulsive disorder and substance use disorder: a new heuristic. Hum Psychopharmacol. 2014;29(1):89-93. doi:10.1002/hup.2373 24424710

[18] Regier DA, Farmer ME, Rae DS, . Comorbidity of mental disorders with alcohol and other drug abuse: results from the Epidemiologic Catchment Area (ECA) Study. JAMA. 1990;264(19):2511-2518. doi:10.1001/jama.1990.03450190043026 2232018

[19] Blom RM, Koeter M, van den Brink W, de Graaf R, Ten Have M, Denys D. Co-occurrence of obsessive-compulsive disorder and substance use disorder in the general population. Addiction. 2011;106(12):2178-2185. doi:10.1111/j.1360-0443.2011.03559.x 21714825

[20] Torres AR, Prince MJ, Bebbington PE, . Obsessive-compulsive disorder: prevalence, comorbidity, impact, and help-seeking in the British National Psychiatric Morbidity Survey of 2000. Am J Psychiatry. 2006;163(11):1978-1985. doi:10.1176/ajp.2006.163.11.1978 17074950

[21] Osland S, Arnold PD, Pringsheim T. The prevalence of diagnosed obsessive compulsive disorder and associated comorbidities: a population-based Canadian study. Psychiatry Res. 2018;268:137-142. doi:10.1016/j.psychres.2018.07.018 30025284

[22] Adam Y, Meinlschmidt G, Gloster AT, Lieb R. Obsessive-compulsive disorder in the community: 12-month prevalence, comorbidity and impairment. Soc Psychiatry Psychiatr Epidemiol. 2012;47(3):339-349. doi:10.1007/s00127-010-0337-5 21287144

[23] Polderman TJ, Benyamin B, de Leeuw CA, . Meta-analysis of the heritability of human traits based on fifty years of twin studies. Nat Genet. 2015;47(7):702-709. doi:10.1038/ng.3285 25985137

[24] Mataix-Cols D, Boman M, Monzani B, . Population-based, multigenerational family clustering study of obsessive-compulsive disorder. JAMA Psychiatry. 2013;70(7):709-717. doi:10.1001/jamapsychiatry.2013.3 23699935

[25] Iervolino AC, Rijsdijk FV, Cherkas L, Fullana MA, Mataix-Cols D. A multivariate twin study of obsessive-compulsive symptom dimensions. Arch Gen Psychiatry. 2011;68(6):637-644. doi:10.1001/archgenpsychiatry.2011.54 21646580

[26] Nurnberger JI Jr, Wiegand R, Bucholz K, . A family study of alcohol dependence: coaggregation of multiple disorders in relatives of alcohol-dependent probands. Arch Gen Psychiatry. 2004;61(12):1246-1256. doi:10.1001/archpsyc.61.12.1246 15583116

[27] Abdellaoui A, Smit DJA, van den Brink W, Denys D, Verweij KJH. Genomic relationships across psychiatric disorders including substance use disorders. Drug Alcohol Depend. 2021;220:108535. doi:10.1016/j.drugalcdep.2021.108535 33524898

[28] Strom NI, Yu D, Gerring ZF, . Genome-wide association study identifies new locus associated with OCD. medRxiv. Preprint posted online October 23, 2021. doi:10.1101/2021.10.13.21261078

[29] Ludvigsson JF, Otterblad-Olausson P, Pettersson BU, Ekbom A. The Swedish personal identity number: possibilities and pitfalls in healthcare and medical research. Eur J Epidemiol. 2009;24(11):659-667. doi:10.1007/s10654-009-9350-y 19504049 PMC2773709

[30] Ludvigsson JF, Andersson E, Ekbom A, . External review and validation of the Swedish National Inpatient Register. BMC Public Health. 2011;11(1):450. doi:10.1186/1471-2458-11-450 21658213 PMC3142234

[31] Brottsförebyggande rådet [Swedish National Council for Crime Prevention]. Swedish Crime Statistics. 2020. Accessed March 31, 2022. https://bra.se/bra-in-english/home.html

[32] Brooke HL, Talbäck M, Hörnblad J, . The Swedish Cause of Death Register. Eur J Epidemiol. 2017;32(9):765-773. doi:10.1007/s10654-017-0316-1 28983736 PMC5662659

[33] Ludvigsson JF, Almqvist C, Bonamy AK, . Registers of the Swedish total population and their use in medical research. Eur J Epidemiol. 2016;31(2):125-136. doi:10.1007/s10654-016-0117-y 26769609

[34] Ekbom A. The Swedish Multi-Generation Register. Methods Mol Biol. 2011;675:215-220. doi:10.1007/978-1-59745-423-0_10 20949391

[35] Anckarsäter H, Lundström S, Kollberg L, . The Child and Adolescent Twin Study in Sweden (CATSS). Twin Res Hum Genet. 2011;14(6):495-508. doi:10.1375/twin.14.6.495 22506305

[36] Rück C, Larsson KJ, Lind K, . Validity and reliability of chronic tic disorder and obsessive-compulsive disorder diagnoses in the Swedish National Patient Register. BMJ Open. 2015;5(6):e007520. doi:10.1136/bmjopen-2014-007520 26100027 PMC4480012

[37] Bergman D, Hagström H, Capusan AJ, . Incidence of *ICD*-based diagnoses of alcohol-related disorders and diseases from Swedish nationwide registers and suggestions for coding. Clin Epidemiol. 2020;12:1433-1442. doi:10.2147/CLEP.S285936 33408530 PMC7781026

[38] Bejerot S, Edman G, Anckarsäter H, . The Brief Obsessive-Compulsive Scale (BOCS): a self-report scale for OCD and obsessive-compulsive related disorders. Nord J Psychiatry. 2014;68(8):549-559. doi:10.3109/08039488.2014.884631 24568661 PMC4221004

[39] Krebs G, Mataix-Cols D, Rijsdijk F, . Concurrent and prospective associations of obsessive-compulsive symptoms with suicidality in young adults: a genetically-informative study. J Affect Disord. 2021;281:422-430. doi:10.1016/j.jad.2020.10.065 33359955 PMC7843953

[40] Saunders JB, Aasland OG, Babor TF, de la Fuente JR, Grant M. Development of the Alcohol Use Disorders Identification Test (AUDIT): WHO collaborative project on early detection of persons with harmful alcohol consumption-II. Addiction. 1993;88(6):791-804. doi:10.1111/j.1360-0443.1993.tb02093.x 8329970

[41] Doyle SR, Donovan DM, Kivlahan DR. The factor structure of the Alcohol Use Disorders Identification Test (AUDIT). J Stud Alcohol Drugs. 2007;68(3):474-479. doi:10.15288/jsad.2007.68.474 17446988

[42] Berman AH, Bergman H, Palmstierna T, Schlyter F. Evaluation of the Drug Use Disorders Identification Test (DUDIT) in criminal justice and detoxification settings and in a Swedish population sample. Eur Addict Res. 2005;11(1):22-31. doi:10.1159/000081413 15608468

[43] Hildebrand M. The psychometric properties of the Drug Use Disorders Identification Test (DUDIT): a review of recent research. J Subst Abuse Treat. 2015;53:52-59. doi:10.1016/j.jsat.2015.01.008 25682718

[44] Birmaher B, Khetarpal S, Brent D, . The Screen for Child Anxiety Related Emotional Disorders (SCARED): scale construction and psychometric characteristics. J Am Acad Child Adolesc Psychiatry. 1997;36(4):545-553. doi:10.1097/00004583-199704000-00018 9100430

[45] Hale WW III, Raaijmakers Q, Muris P, Meeus W. Psychometric properties of the Screen for Child Anxiety Related Emotional Disorders (SCARED) in the general adolescent population. J Am Acad Child Adolesc Psychiatry. 2005;44(3):283-290. doi:10.1097/00004583-200503000-00013 15725973

[46] Monga S, Birmaher B, Chiappetta L, . Screen for Child Anxiety-Related Emotional Disorders (SCARED): convergent and divergent validity. Depress Anxiety. 2000;12(2):85-91. doi:10.1002/1520-6394(2000)12:2<85::AID-DA4>3.0.CO;2-2 11091931

[47] Kohout FJ, Berkman LF, Evans DA, Cornoni-Huntley J. Two shorter forms of the CES-D (Center for Epidemiological Studies Depression) depression symptoms index. J Aging Health. 1993;5(2):179-193. doi:10.1177/089826439300500202 10125443

[48] Carpenter JS, Andrykowski MA, Wilson J, . Psychometrics for two short forms of the Center for Epidemiologic Studies–Depression Scale. Issues Ment Health Nurs. 1998;19(5):481-494. doi:10.1080/016128498248917 9782864

[49] Verhulst B, Prom-Wormley E, Keller M, Medland S, Neale MC. Type I error rates and parameter bias in multivariate behavioral genetic models. Behav Genet. 2019;49(1):99-111. doi:10.1007/s10519-018-9942-y 30569348 PMC6345547

[50] Rijsdijk FV, Sham PC. Analytic approaches to twin data using structural equation models. Brief Bioinform. 2002;3(2):119-133. doi:10.1093/bib/3.2.119 12139432

[51] Plana-Ripoll O, Pedersen CB, Holtz Y, . Exploring comorbidity within mental disorders among a Danish national population. JAMA Psychiatry. 2019;76(3):259-270. doi:10.1001/jamapsychiatry.2018.3658 30649197 PMC6439836

[52] Virtanen S, Kuja-Halkola R, Mataix-Cols D, . Comorbidity of substance misuse with anxiety-related and depressive disorders: a genetically informative population study of 3 million individuals in Sweden. Psychol Med. 2020;50(10):1706-1715. doi:10.1017/S0033291719001788 31328718

[53] Conway KP, Swendsen J, Husky MM, He JP, Merikangas KR. Association of lifetime mental disorders and subsequent alcohol and illicit drug use: results from the National Comorbidity Survey–Adolescent Supplement. J Am Acad Child Adolesc Psychiatry. 2016;55(4):280-288. doi:10.1016/j.jaac.2016.01.006 27015718

[54] Groenman AP, Janssen TWP, Oosterlaan J. Childhood psychiatric disorders as risk factor for subsequent substance abuse: a meta-analysis. J Am Acad Child Adolesc Psychiatry. 2017;56(7):556-569. doi:10.1016/j.jaac.2017.05.004 28647007

[55] Gage SH, Davey Smith G, Ware JJ, Flint J, Munafò MR. G = E: what GWAS can tell us about the environment. PLoS Genet. 2016;12(2):e1005765. doi:10.1371/journal.pgen.1005765 26866486 PMC4750859

[56] Arnold PD, Askland KD, Barlassina C, ; International Obsessive Compulsive Disorder Foundation Genetics Collaborative (IOCDF-GC) and OCD Collaborative Genetics Association Studies (OCGAS). Revealing the complex genetic architecture of obsessive-compulsive disorder using meta-analysis. Mol Psychiatry. 2018;23(5):1181-1188. doi:10.1038/mp.2017.154 28761083 PMC6660151

[57] Bakhshaie J, Storch EA, Zvolensky MJ. Obsessive-compulsive symptoms and problematic alcohol use: the explanatory role of drinking motives. Addict Behav. 2021;115:106734. doi:10.1016/j.addbeh.2020.106734 33385756 PMC12818886

[58] Müller CP, Homberg JR. The role of serotonin in drug use and addiction. Behav Brain Res. 2015;277:146-192. doi:10.1016/j.bbr.2014.04.007 24769172

[59] Torrens M, Fonseca F, Mateu G, Farré M. Efficacy of antidepressants in substance use disorders with and without comorbid depression: a systematic review and meta-analysis. Drug Alcohol Depend. 2005;78(1):1-22. doi:10.1016/j.drugalcdep.2004.09.004 15769553

[60] Virtanen S, Lagerberg T, Khemiri L, . Association of selective serotonin re-uptake inhibitor (SSRI) treatment with acute substance misuse outcomes. Addiction. 2022;117(1):234-242. doi:10.1111/add.15625 34185347

[61] Abramowitz JS, Fabricant LE, Taylor S, Deacon BJ, McKay D, Storch EA. The relevance of analogue studies for understanding obsessions and compulsions. Clin Psychol Rev. 2014;34(3):206-217. doi:10.1016/j.cpr.2014.01.004 24561743

