# Supplementary material for: Association of Obsessive-Compulsive Disorder and Obsessive-Compulsive Symptoms With Substance Misuse in 2 Longitudinal Cohorts in Sweden
Source: JAMA Netw Open. 2022 Jun 6;5(6):e2214779. doi: 10.1001/jamanetworkopen.2022.14779 (PMC9171556; doi:10.1001/jamanetworkopen.2022.14779)
Supplement: Supplement. — eMethods. Post hoc Comorbidity Analyses eTable 1. ICD-10 Codes for Substance Use-Related Disorders and Deaths eTable 2. Population Cohort Characteristics eTable 3. Post hoc Analyses: Association of Obsessive-Compulsive Disorder With Substance Misuse Outcomes, Adjusted for Psychiatric Comorbidities eTable 4. Descriptive Statistics of the Child and Adolescent Twin Study in Sweden (CATSS) Twin Cohort eTable 5. Population Cohort: Fit Statistics From the Quantitative Genetic Models eTable 6. Population Cohort: Parameter Estimates (95% CIs) From the Full ACE-ACE Model and the Best-Fitting Bivariate Model eTable 7. CATSS Cohort: Fit Statistics From the Quantitative Genetic Models eTable 8. CATSS Cohort: Parameter Estimates (95% CIs) From the Full ACE-ACE Model and the Best-Fitting Bivariate Model [file jamanetwopen-e2214779-s001.pdf]

Supplementary Online Content

Virtanen S, Kuja-Halkola R, Sidorchuk A, et al. Association of obsessive-compulsive disorder and obsessive-compulsive symptoms with substance misuse in 2 longitudinal cohorts in Sweden. *JAMA Netw Open*. 2022;5(6):e2214779. doi:10.1001/jamanetworkopen.2022.14779

eMethods. Post hoc Comorbidity Analyses
eTable 1. *ICD-10* Codes for Substance Use-Related Disorders and Deaths
eTable 2. Population Cohort Characteristics
eTable 3. Post hoc Analyses: Association of Obsessive-Compulsive Disorder With Substance Misuse Outcomes, Adjusted for Psychiatric Comorbidities
eTable 4. Descriptive Statistics of the Child and Adolescent Twin Study in Sweden (CATSS) Twin Cohort
eTable 5. Population Cohort: Fit Statistics From the Quantitative Genetic Models
eTable 6. Population Cohort: Parameter Estimates (95% CIs) From the Full ACE-ACE Model and the Best-Fitting Bivariate Model
eTable 7. CATSS Cohort: Fit Statistics From the Quantitative Genetic Models
eTable 8. CATSS Cohort: Parameter Estimates (95% CIs) From the Full ACE-ACE Model and the Best-Fitting Bivariate Model

This supplementary material has been provided by the authors to give readers additional information about their work.

© 2022 Virtanen S et al. *JAMA Network Open*.

eMethods. Post hoc Comorbidity Analyses

We collected lifetime (registered between 1997-2013) ICD-10 diagnoses for other psychiatric comorbidities from the National Patient register. Diagnoses included attention-deficit/hyperactivity disorder (ICD code: F90), autism spectrum disorder (F841, F845, F848, F849), bipolar disorder (F30-F31, F25.0, F34.0), psychotic disorders (F21-F24, F25 excluding F25.0, F28-F29), any personality disorder (F60), dissocial personality disorder (F60.2), and conduct disorders (F91).

To test whether the association between OCD and substance misuse was explained by psychiatric comorbidities other that anxiety and depressive disorders, we estimated their association adjusting for these additional psychiatric comorbidities, one at the time.

© 2022 Virtanen S et al. *JAMA Network Open*.

eTable 1. *ICD-10* Codes for Substance Use-Related Disorders and Deaths

**Substance**	**Mental and Behavioral Disorders**	**Poisoning**	**Somatic Conditions**	**Other Related Codes**
**Alcohol**	F10	F51.0	E24.4	035.4
			G31.2	X65
			G62.1	Y57.3
			G72.1	Y90
			I42.6	Y91
			K29.2	Z50.2
			K70.0	Z71.4
			K70.1	Z72.1
			K70.2	
			K70.3	
			K70.4	
			K70.9	
			K85.2	
			K86.0	
**Opioids**	F11	T40.0		
		T40.1		
		T40.2		
		T40.3		
**Cannabis**	F12	T40.7		
**Sedatives**	F13	T42.3		
		T42.4		
**Stimulants**	F14	T40.5		
	F15	T40.6		
**Other drugs**	F16	T40.4		
	F18	T40.6		
	F19	T40.8		
		T40.9		

© 2022 Virtanen S et al. *JAMA Network Open*.

eTable 2. Population Cohort Characteristics

	Total population sample, n=6,304,188	Unaffected General Population (n=6,276,846)	Individuals with Obsessive-compulsive disorder (n=27,342)
**Birth year, median**	1966	1966	1980
**Age at start of follow-up, median**	30.5	30.5	16.9
**Sex, n (%)**			
Men	3,224,538 (51.1)	3,212,821 (51.2)	11,717 (42.9)
Women	3,079,650 (48.9)	3,064,025 (48.8)	15,625 (57.2)
**Education, n (%)**			
Primary school	1,283,341 (20.4)	1,276,420 (20.3)	6,921 (25.3)
High school	2,919,036 (46.3)	2,906,902 (46.3)	12,134 (44.4)
University	2,009,023 (31.9)	2,001,323 (31.9)	7,700 (28.1)
Doctorate	54,729 (0.8)	54,628 (0.9)	101 (0.4)
Unknown	38,059 (0.6)	37,573 (0.6)	486 (1.8)
**Parent born abroad[a], n (%)**	985,721 (15.6)	980,964 (15.6)	4,757 (17.4)
**Psychiatric disorders**			
Obsessive-compulsive disorder	27,342 (0.4)	NA	NA
Anxiety disorder	264,890 (4.2)	250,302 (4.0)	14,588 (53.4)
Depressive disorder	315,911 (5.0)	303,016 (4.8)	12,895 (47.2)
Attention-deficit/hyperactivity disorder	77,768 (1.2)	73,953 (1.2)	3,815 (14.0)
Autism spectrum disorder	35,141 (0.6)	31,847 (0.5)	3,294 (12.1)
Bipolar disorder	54,629 (0.9)	51,868 (0.8)	2,761 (10.1)
Psychotic disorder	58,088 (0.9)	55,342 (0.9)	2,746 (10.0)
Any personality disorder	22,489 (0.4)	20,565 (0.3)	1,924 (7.0)
Dissocial personality/conduct disorder	9,405 (0.2)	8,924 (0.1)	481 (1.8)
**Median follow-up length, years**	16.9	16.9	13.7
**Median age at first OCD diagnosis**	NA	NA	28.0

Note: [a] At least one parent born outside Sweden

© 2022 Virtanen S et al. *JAMA Network Open*.

eTable 3. Post hoc Analyses: Association of Obsessive-Compulsive Disorder With Substance Misuse Outcomes, Adjusted for Psychiatric Comorbidities

	HR (95% CI), Adjusted for Sex, Birth Year, and Comorbid Disorder						
**Outcome**	**Without adjustment**	**ADHD**	**Autism Spectrum Disorders**	**Bipolar Disorder**	**Psychotic Disorders**	**Any Personality Disorder**	**Dissocial Personality Disorder or Conduct Disorder**
**Any substance misuse outcome**	3.68 (3.52-3.85)	2.23 (2.13-2.33)	2.99 (2.86-3.13)	2.68 (2.56-2.80)	2.54 (2.43-2.65)	2.44 (2.33-2.55)	3.09 (2.96-3.24)
**Alcohol-related disorders**	4.51 (4.25-4.79)	2.61 (2.46-2.77)	3.53 (3.32-3.75)	2.97 (2.80-3.16)	2.90 (2.73-3.08)	2.49 (2.34-2.65)	3.79 (3.57-4.03)
**Acute alcohol intoxications**	3.64 (3.38-3.93)	2.36 (2.19-2.55)	3.05 (2.82-3.29)	2.61 (2.42-2.82)	2.65 (2.45-2.85)	2.31 (2.14-2.49)	3.14 (2.91-3.38)
**Any drug-related disorders**	6.59 (6.33-7.07)	2.75 (2.61-2.91)	4.37 (4.13-4.62)	3.68 (3.48-3.89)	3.18 (3.01-3.36)	2.90 (2.75-3.07)	4.71 (4.45-4.98)
Opioid-related disorders	6.19 (5.47-6.43)	2.45 (2.18-2.75)	4.33 (3.84-4.88)	3.76 (3.35-4.23)	3.13 (2.78-3.51)	2.41 (2.14-2.71)	4.16 (3.70-4.67)
Cannabis-related disorders	3.72 (3.31-4.32)	1.47 (1.27-1.71)	2.55 (2.19-2.97)	2.34 (2.02-2.71)	1.44 (1.24-1.68)	2.03 (1.75-2.36)	2.47 (2.13-2.87)
Sedative-related disorders	10.53 (9.84-11.28)	4.53 (4.23-4.86)	6.53 (6.08-7.01)	4.84 (4.52-5.19)	5.05 (4.71-5.41)	3.35 (3.12-3.59)	7.54 (7.03-8.09)
Stimulant-related disorders	4.89 (4.25-5.61)	1.39 (1.21-1.59)	3.00 (2.60-3.45)	2.86 (2.48-3.28)	1.64 (1.43-1.89)	1.84 (1.60-2.11)	2.88 (2.51-3.32)
Other drug-related disorders	6.32 (5.86-6.81)	2.22 (2.06-2.40)	3.93 (3.64-4.25)	3.40 (3.15-3.66)	2.49 (2.31-2.69)	2.33 (2.16-2.52)	3.94 (3.65-4.25)
**Substance-related convictions**	1.24 (1.09-1.41)	0.77 (0.67-0.87)	1.21 (1.06-1.37)	1.04 (0.92-1.19)	1.04 (0.91-1.18)	0.96 (0.84-1.09)	1.05 (0.92-1.19)
**Substance-related deaths**	5.20 (4.45-6.08)	4.27 (3.65-5.00)	4.96 (4.24-5.81)	4.49 (3.80-5.21)	3.75 (3.20-4.39)	3.42 (2.92-4.01)	4.65 (3.98-5.44)

© 2022 Virtanen S et al. *JAMA Network Open*.

eTable 4. Descriptive Statistics of the Child and Adolescent Twin Study in Sweden (CATSS) Twin Cohort

	Cohort	
	**CATSS-18 (n=9,230)**	**CATSS-24 (n=3,414)**
**Sex, n (%)**		
Men	3,679 (39.9)	1,266 (37.1)
Women	5,551 (60.1)	2,148 (62.9)
**Birth year, mean (SD)**	1996.8 (2.6)	1994.5 (1.2)
**Measured at age 18**		
Obsessive-compulsive disorder symptoms, mean (SD)	1.9 (2.3)	1.7 (2.1)
Anxiety symptoms, mean (SD)	16.3 (11.6)	15.1 (10.4)
Depressive symptoms, mean (SD)	10.3 (4.7)	10.1 (4.6)
Endorsed using alcohol, n (%)	9,219 (99.9)	2,984 (87.4)
Endorsed using drugs, n (%)	749 (8.1)	205 (6.0)
Alcohol dependence symptoms, mean (SD)[a]	1.6 (2.6)	1.5 (2.5)
Drug dependence symptoms, mean (SD)[a]	2.1 (3.6)	1.8 (3.3)
**Measured at age 24**		
Endorsed using alcohol, n (%)	NA	3,392 (99.4)
Endorsed using drugs, n (%)	NA	453 (13.3)
Alcohol dependence symptoms, mean (SD)[a]	NA	1.4 (2.4)
Drug dependence symptoms, mean (SD)[a]	NA	2.2 (4.1)

Note: [a] Means for alcohol and drug dependence symptoms where calculated for participants who endorsed using alcohol and drugs, respectively.

The sample from the age 18 assessment included 2,056 monozygotic and 2,250 dizygotic twins whose co-twin also participated in the study. There were 3,277 opposite-sex dizygotic twins, 221 twins with unknown zygosity, and 524 monozygotic and 902 dizygotic twins without their co-twin in the study, who were excluded from the quantitative genetic analyses, but contributed to the phenotypic analyses.

© 2022 Virtanen S et al. *JAMA Network Open*.

eTable 5. Population Cohort: Fit Statistics From the Quantitative Genetic Models

Model	Components[a,b]	Ep[c]	Minus2LL	Df[d]	AIC[e]	p-value (compared to ACE-ACE)
1	ACE-ACE	17	1632456.9	6894833	-12157209	NA
2	AE-ACE	15	1632457.3	6894835	-12157213	.80
3	AE-AE	14	1632460.9	6894836	-12157211	.26

Note: [a] Components=Estimated variance components for OCD (1st variable) and substance misuse (2nd variable)
[b] A=Additive genetic factors, C=Shared environmental factors, E=Non-shared environmental factors
[c] Ep=Number of estimated parameters
[d] Df=Degrees of freedom
[e] AIC=Akaike Information Criterion

© 2022 Virtanen S et al. *JAMA Network Open*.

eTable 6. Population Cohort: Parameter Estimates (95% CIs) From the Full ACE-ACE Model and the Best-Fitting Bivariate Model

Model	Variance Components[b]			Genetic and Environmental Correlations[c]			Contributions to Covariance		
	A	C	E	r_A	r_C	r_E	Bivariate A	Bivariate C	Bivariate E
**Model A: ACE bivariate model between OCD and substance misuse**									
OCD	0.67 (0.21, 1.18)	-0.06 (-0.31, 0.17)	0.39 (0.13, 0.63)	0.21 (-0.05, 0.48)	NA[a]	0.32 (0.12, 0.51)	0.45 (-0.14, 1.0.4)	0.05 (-0.23, 0.34)	0.50 (0.19, 0.80)
Substance misuse	0.49 (0.40, 0.57)	0.04 (0, 0.08)	0.47 (0.43, 0.52)						
**Model B: AE bivariate model between OCD and substance misuse**									
OCD	0.55 (0.50, 0.59)	NA	0.45 (0.41, 0.50)	0.28 (0.24, 0.32)	NA[a]	0.27 (0.22, 0.32)	0.56 (0.49, 0.64)	NA	0.44 (0.36, 0.51)
Substance misuse	0.57 (0.56, 0.58)	NA	0.43 (0.42, 0.44)						

Note: [a] r_C could not be estimated because of the negative shared environmental variance of OCD
[b] A=Additive genetic factors; C=Shared environmental factors; E=Non-shared environmental factors;
[c] r =correlation between variance components.
All presented confidence intervals are Wald-type confidence intervals. Because of the direct-symmetric parameterization and the use of Wald-type confidence intervals, variance component estimates can include negative values, and CIs may span outside the parameter space.

© 2022 Virtanen S et al. *JAMA Network Open*.

eTable 7. CATSS Cohort: Fit Statistics From the Quantitative Genetic Models

Model	Components[a,b]	Ep[c]	Minus2LL	Df[d]	AIC[e]	p-value (compared to ACE-ACE)
1	ACE-ACE	11	24335.53	11437	1461.53	NA
2	AE-ACE	9	24342.73	11439	1464.73	.03
3	AE-AE	8	24342.98	11440	1462.98	.06

Note: [a] Components=Estimated variance components for OCD (1st variable) and substance misuse (2nd variable)
[b] A=Additive genetic factors, C=Shared environmental factors, E=Non-shared environmental factors
[c] Ep=Number of estimated parameters
[d] Df=Degrees of freedom
[e] AIC=Akaike Information Criterion

© 2022 Virtanen S et al. *JAMA Network Open*.

eTable 8. CATSS Cohort: Parameter Estimates (95% CIs) From the Full ACE-ACE Model and the Best-Fitting Bivariate Model

Model	Variance Components[b]			Genetic and Environmental Correlations[c]			Contributions to Covariance		
	A	C	E	r_A	r_C	r_E	Bivariate A	Bivariate C	Bivariate E
**Model A: ACE bivariate model between OCD symptoms and alcohol dependence symptoms**									
OCD symptoms	0.51 (0.36, 0.66)	-0.13 (-0.25, 0)	0.62 (0.57, 0.67)	0.14 (-0.09, 0.36)	NA[a]	0.12 (0.06, 0.18)	0.33 (-0.24, 0.88)	0.31 (-0.16, 0.77)	0.37 (0.19, 0.55)
Alcohol dependence	0.40 (0.26, 0.55)	0.05 (-0.07, 0.17)	0.54 (0.50, 0.59)						
**Model B: AE bivariate model between OCD symptoms and alcohol dependence symptoms**									
OCD symptoms	0.36 (0.31, 0.41)	NA	0.64 (0.59, 0.69)	0.31 (0.23, 0.40)	NA[a]	0.10 (0.05, 0.16)	0.68 (0.51, 0.84)	NA	0.32 (0.16, 0.49)
Alcohol dependence	0.46 (0.42, 0.51)	NA	0.54 (0.49, 0.58)						

Note: [a] r_C could not be estimated because of the negative shared environmental variance of OCD

[b] A=Additive genetic factors; C=Shared environmental factors; E=Non-shared environmental factors;

[c] r =correlation between variance components.

All presented confidence intervals are Wald-type confidence intervals. Because of the direct-symmetric parameterization and the use of Wald-type confidence intervals, variance component estimates can include negative values, and CIs may span outside the parameter space

© 2022 Virtanen S et al. *JAMA Network Open*.
